# Divergent hemispheric reasoning strategies: reducing uncertainty versus resolving inconsistency

**DOI:** 10.3389/fnhum.2014.00839

**Published:** 2014-10-21

**Authors:** Nicole Marinsek, Benjamin O. Turner, Michael Gazzaniga, Michael B. Miller

**Affiliations:** ^1^Dynamical Neuroscience, University of CaliforniaSanta Barbara, CA, USA; ^2^Psychological and Brain Sciences, University of CaliforniaSanta Barbara, CA, USA

**Keywords:** reasoning, split-brain, lateralization, delusions, inference making

## Abstract

Converging lines of evidence from diverse research domains suggest that the left and right hemispheres play distinct, yet complementary, roles in inferential reasoning. Here, we review research on split-brain patients, brain-damaged patients, delusional patients, and healthy individuals that suggests that the left hemisphere tends to create explanations, make inferences, and bridge gaps in information, while the right hemisphere tends to detect conflict, update beliefs, support mental set-shifts, and monitor and inhibit behavior. Based on this evidence, we propose that the left hemisphere specializes in creating hypotheses and representing causality, while the right hemisphere specializes in evaluating hypotheses, and rejecting those that are implausible or inconsistent with other evidence. In sum, we suggest that, in the domain of inferential reasoning, the left hemisphere strives to reduce uncertainty while the right hemisphere strives to resolve inconsistency. The hemispheres’ divergent inferential reasoning strategies may contribute to flexible, complex reasoning in the healthy brain, and disruption in these systems may explain reasoning deficits in the unhealthy brain.

## Introduction

Hemispheric specialization has become a widely accepted principle of cortical organization. For higher-order association areas such as the prefrontal cortex (PFC), this is probably both a consequence of the hemispheric specialization of the lower-order regions that constitute the predominant inputs to these regions (Craig, 2005), and also a response to the evolutionary pressure against redundant functionality given limited cortical space (Corballis, 1989; Gazzaniga, 2000; Vallortigara, 2006; Hopkins and Cantalupo, 2008). However, most previous theories of hypothesis formation and evaluation have focused on other topographical distinctions—for example, between the dorsal and ventral lateral regions of the PFC (Barbey and Patterson, 2011). Here, we present evidence drawn from multiple distinct domains and a variety of experimental paradigms to demonstrate that the right and left hemispheres have distinctive strengths and weaknesses in inferential reasoning.

Several distinct lines of evidence indicate that each hemisphere plays a unique role in inference making. Perhaps the strongest line of evidence comes from Michael Gazzaniga’s split-brain work, which provides a unique view into the operation of each hemisphere in isolation. Research on patients with unilateral brain damage also sheds light on the sufficiency and necessity of each hemisphere for various processes. Additionally, some psychiatric conditions that involve failures in inference making have been linked to hemispheric differences; delusions in particular reveal a strong link between hemispheric changes and aberrant reasoning. And finally, within the last two decades, cognitive neuroscience research using largely healthy populations has provided substantially more insight into some of the various component processes that comprise successful inferential reasoning.

In this review, we describe and categorize hemispheric biases in inferential reasoning. Although we may discuss each hemisphere as a whole, we acknowledge that reasoning strategies arise from processing biases in one or many of the specialized modules that make up each hemisphere. That is, the properties of the localized neural processes in each hemisphere combine to create divergent reasoning strategies at the hemispheric level but may not generalize to all modules in the hemisphere. Although this localized processing view is more accurate, at times we simply refer to each hemisphere, rather than the neural processes that comprise it, for the sake of brevity.

This review is structured to highlight the dominant contributions of each hemisphere in inferential reasoning. The first two sections describe the various processes that each hemisphere excels at. We do not have separate sections highlighting each hemisphere’s weaknesses, because unless noted otherwise, the hemispheres act as foils for one another—the strengths of one hemisphere constitute the weaknesses of the other. Taken together, there is a common thread that ties together the unique abilities of each hemisphere, which we further develop into a qualitative theory for how the two hemispheres work together to perform inferential reasoning in the intact brain. To preview, there is substantial evidence that the left hemisphere tends to form hypotheses and the right hemisphere tends to evaluate and revise them.

Although a great deal of evidence from a broad cross-section of the psychological literature is consistent with this framework, we are careful to highlight those studies whose results contradict our hypotheses. There are also a number of issues that obfuscate this area of research, which we discuss in a final section on problems and challenges.

## Left hemisphere processes

### Explanation

Ample evidence suggests that the left hemisphere creates explanations when faced with uncertainty or ambiguity, and split-brain studies offer especially salient examples of the left hemisphere’s proclivity to explain (Gazzaniga, 1989, 2000). In split-brain patients, the corpus callosum—the large bundle of axons connecting the two hemispheres—is severed as a treatment for intractable epilepsy. Since the two hemispheres of a split-brain patient are disconnected and independent of one another, researchers can study the cognitive abilities of each hemisphere in isolation.

In one well-known experiment, a split-brain patient’s left hemisphere was shown a picture of a chicken claw and his right hemisphere was shown a picture of a snow scene. The patient was asked to point to a card that was associated with the picture he just saw. With his left hand (controlled by his right hemisphere) he selected a shovel, which matched the snow scene. With his right hand (controlled by his left hemisphere) he selected a chicken, which matched the chicken claw. Next, the experimenter asked the patient why he selected each item. One would expect the speaking left hemisphere to explain why it chose the chicken but *not* why it chose the shovel, since the left hemisphere did not have access to information about the snow scene. Instead, the patient’s speaking left hemisphere replied, “Oh, that’s simple. The chicken claw goes with the chicken and you need a shovel to clean out the chicken shed” (Gazzaniga, 2000). The left hemisphere quickly and confidently created an explanation for the behavior—an explanation that was incorrect but nonetheless plausible, given the left hemisphere’s limited information. In another experiment, researchers instructed the right hemisphere of a split-brain patient to stand. After the patient stood, experimenters asked the patient why he did so. Again, instead of admitting that he did not know why he stood, the speaking left hemisphere created an explanation, insisting he was thirsty and wanted a drink (see Gazzaniga and Miller, 2009).

In these cases, the patients’ left hemispheres created explanations that adequately accounted for their behaviors. Even though the explanations were wrong, they were rational and plausible, given the available evidence. When evidence is sparse or unusual, however, the left hemisphere may create seemingly bizarre and implausible explanations, which may develop into delusions. Delusions are defined as “fixed beliefs that are not amenable to change in light of conflicting evidence” (American Psychiatric Association, 2013). Coltheart et al. propose that delusions arise when (1) aberrant evidence prompts an explanation; and (2) an evaluative system fails to reject implausible explanations (Coltheart, 2010; Coltheart et al., 2010, 2011). Delusions may also arise when the left hemisphere’s tendency to explain goes awry; indeed, delusional disorders are characterized by excessive inference making (Braun and Suffren, 2011) and the tendency to prematurely jump to conclusions (Huq et al., 1988; Dudley et al., 1997; Conway et al., 2002; Moritz and Woodward, 2005; Warman et al., 2007). Importantly, delusions have consistently been linked to both right hemisphere damage (Devinsky, 2009; Coltheart, 2010; Braun and Suffren, 2011) and left hemisphere overactivity (Leutmezer et al., 2003; Mucci et al., 2005; *cf* Luat et al., 2008; Braun and Suffren, 2011). Together, these lines of evidence suggest that the left hemisphere creates explanations that best account for available data. The left hemisphere’s ability to explain, however, is only as good as the information it receives: if it reasons with incomplete or ambiguous evidence, it may create explanations that are incorrect, inappropriate, or even bizarre (Cooney and Gazzaniga, 2003).

Research on healthy subjects also indicates that the left hemisphere plays a prominent role in explanation. Parris et al. (2009) recorded subjects’ brain activity with fMRI as they watched videos of magic tricks and control videos, which had the same beginning as the magic trick videos but ended with either an expected or unrelated surprising event. They found that only the left dorsolateral PFC was more active when subjects viewed magic tricks than when they viewed expected or surprising events. As the researchers concluded, this increased activity may reflect the left hemisphere’s attempt to explain the magic trick.

### Filling in gaps

The left hemisphere appears to detest uncertainty; it creates explanations and fills in gaps of information in order to build a cohesive story and extinguish doubt. That is, in addition to constructing an explanatory hypothesis for the evidence it encounters, the left hemisphere seems to spontaneously generate predictions in accordance with its hypothesis (note that while this is consistent with the increasingly popular notion that the brain is fundamentally a prediction engine (e.g., Bar, 2007), we are limiting our scope here to inferential reasoning). This propensity to fill in gaps may become excessive and lead to errors in reasoning. Goel et al. (2007) found that the left hemisphere can correctly form simple inferences, but tends to *over* interpret ambiguous relationships: it draws conclusions with incomplete information and infers relationships where there are none.

The left hemisphere’s tendency to fill in gaps is also apparent in recognition memory tests. The left hemisphere often creates errors of commission—it falsely remembers seeing items that were not presented before (Phelps and Gazzaniga, 1992; Metcalfe et al., 1995; Braun, 2007; Braun et al., 2009; but *cf*. Stuss and Alexander, 2007). Phelps and Gazzaniga (1992) presented visual scenes with a common theme to split-brain patients and later tested each hemisphere’s memory for the scenes. During the recognition test, the left hemisphere falsely remembered scenes that were not present in the study phase, but that fit the gist of the presented scenes. The right hemisphere, on the other hand, demonstrated a veridical memory for the scenes. Again, the results suggest that the left hemisphere bridged semantic gaps, which led to the false recognition of items that fit a schema or gist.

Studies on discourse comprehension and inference making in language also suggest the left hemisphere bridges semantic gaps. Specifically, the left hemisphere has been shown to draw coherence inferences, or contextually relevant connections between intermediately related material. Lesion studies show that patients with right hemisphere damage retain the ability to draw coherence inferences (Tompkins et al., 2004; but *cf*. Beeman, 1993, which reports mixed results). Similarly, several neuroimaging studies have reported left-dominant activity specifically when participants draw bridging inferences, or encounter information that is only intermediately related to context (Kuperberg et al., 2006; Virtue et al., 2006; Friese et al., 2008). Finally, priming studies in healthy individuals supports left hemisphere (to a numerically larger degree than right hemisphere) involvement in inferencing (Beeman et al., 2000; Powers et al., 2012, for narratives but not conversations).

### Inference making

Patients with an intact left hemisphere, but damaged right hemisphere, retain the ability to make simple inferences about hypothetical situations (Deglin and Kinsbourne, 1996; Caplan and Dapretto, 2001; Ferstl et al., 2002; Goel et al., 2007; Reverberi et al., 2009). Neuroimaging studies using healthy participants also implicate the left hemisphere in inference making: left-lateralized brain networks have been identified for tasks involving interpreting and reasoning about past events (D’Argembeau et al., 2013), making inferences about related sentences (Ferstl and von Cramon, 2002; Friese et al., 2008), and inferring a rule or relationship (Rodriguez-Moreno and Hirsch, 2009).

Additionally, only the left hemisphere can infer causal relationships between two objects: Gazzaniga and Smylie (1984) presented object pairs to one hemisphere of split-brain patients and asked the patients to point to a picture that depicted the outcome of combining the two objects with their contralateral hand. For example, if pictures of a match and wooden log were presented to the left hemisphere, the patient could point to a bonfire (the correct causal outcome), a woodpile, or a lit cigarette with their right hand. Even though both hemispheres possessed a sophisticated lexicon, only the left hemisphere could infer the causal relationship between the two items, and this trend held for both visual and verbal stimuli. The left hemisphere, but not the right, can also use evidence to extract an underlying causal structure (Roser et al., 2005) and may attempt to do so, even when events are random (Wolford et al., 2000).

## Right hemisphere processes

### Conflict detection

The right hemisphere plays a prominent role in detecting inconsistencies between hypotheses and reality. Patients with an intact right hemisphere, but damaged or suppressed left hemisphere, retain the ability to identify semantic inconsistencies (Ferstl et al., 2002) and detect conflict between logic and real-world knowledge (Deglin and Kinsbourne, 1996).

Neuroimaging studies using healthy and deluded individuals also suggest the right hemisphere detects conflict. Studies on healthy subjects consistently find that the right lateral PFC is recruited when logic conflicts with prior beliefs (Goel et al., 2000; Goel and Dolan, 2003; Goel, 2007; Menenti et al., 2009; Stollstorff et al., 2012) and its activity is modulated by the degree to which reasoning problems conflict with real-world knowledge (Stollstorff et al., 2012). Regions in the right hemisphere are also active when subjects receive negative feedback indicating that their hypothesis is no longer correct (Konishi et al., 2002).

Returning to the topic of delusions, these disordered patterns of thinking are characterized not only by excessive inference making, but also the failure to evaluate and update beliefs in accordance with conflicting evidence (Marcel et al., 2004; Coltheart, 2010; Coltheart et al., 2010, 2011). It is possible that the failure to update inappropriate beliefs results from an impaired conflict detection system. In one study, Corlett et al. (2007) taught delusional patients and controls associative mappings between foods and allergic outcomes. After the participants learned the mappings thoroughly, the researchers gave subjects negative feedback that contradicted the mappings they were taught. In healthy controls, the right ventrolateral prefrontal cortex (vlPFC) demonstrated a reward-prediction error: relative to baseline, its activity increased in response to unexpected feedback and decreased in response to expected feedback. The right vlPFC of delusional patients, however, failed to distinguish between expected and unexpected feedback, suggesting the delusional patients’ prediction-error processing was impaired.

### Hypothesis rejection

The right hemisphere may also support the rejection of inappropriate beliefs and hypotheses. When the right hemisphere is damaged, patients’ hypotheses become rigid and impermeable to conflicting evidence. In one experiment, Brownell et al. (1986) showed patients with unilateral right hemisphere brain damage two sentences that could be integrated to form one interpretation. Importantly, one of the two sentences supported a different, incorrect interpretation when presented in isolation. Brownell manipulated the position of the misleading sentence to test the patients’ ability to revise an initial incorrect hypothesis. Compared to controls, patients with right hemisphere brain damage made fewer correct inferences. They could accurately extract true causal relationships but tended to make inappropriate associations between non-related items. Patients’ erroneous inferences were especially prevalent when the misleading information was presented first. These findings suggest that a functioning right hemisphere is necessary to detect inconsistencies and update flawed hypotheses accordingly.

In a similar study, Miller et al. (2010) presented moral reasoning problems to split-brain patients. The problems consisted of scenarios that featured characters with either cruel or helpful intentions and outcomes that were either harmful or neutral. Their results are consistent with the idea that the split-brain patients’ left hemispheres formed quick-and-dirty inferences when presented with initial evidence about the scenario (the potential danger of the situation), and these inferences were resistant to revision when additional evidence about the characters’ intentions was provided. This suggests that, without the right hemisphere’s ability to reject inappropriate or improbable hypotheses when contradictory evidence arises, the left hemisphere maintains its original explanations, even if they are no longer relevant.

Research on delusional patients also strongly implicates the right hemisphere in belief evaluation and revision. As we have noted previously, delusions persist because patients fail to update inappropriate beliefs (Marcel et al., 2004; Coltheart, 2010; Coltheart et al., 2010, 2011) and this failure is rooted in right hemisphere brain damage (Stone et al., 1993; Ramachandran, 1996; Devinsky, 2009; Coltheart, 2010; Coltheart et al., 2010, 2011; Braun and Suffren, 2011). Marcel et al. (2004) examined the beliefs of 64 patients with anosognosia for hemiplegia (denial of paralysis), 44 of whom had unilateral right hemisphere damage and 22 of whom had unilateral left hemisphere damage. They found that patients with right hemisphere lesions, but not so much those with left hemisphere lesions, consistently overestimated their abilities to perform tasks requiring the use of their paralyzed limbs. Moreover, *only* patients with right hemisphere brain damage continued to overestimate their ability to perform a task after they had *just failed* to perform the task in question.

Coltheart et al. (2010) review cases of patients with neurological symptoms that mirror those of delusional patients, but who nonetheless fail to develop delusional beliefs. They propose that these patients, unlike delusional patients, have an intact right frontal lobe, which allows them to reject implausible hypotheses and update their beliefs in accordance with reality. In a particularly striking example in support of this view, Bisiach et al. (1991) temporarily increased activity in the right hemisphere of a patient who believed her left arm belonged to her mother by irrigating her left ear with cold water. Incredibly, the irrigation abolished the patient’s delusion: up to 2 h after the procedure, she rightfully claimed her arm as her own. This finding has since been replicated (Ramachandran, 1996) and supports the necessity of the right hemisphere in updating inappropriate beliefs.

### Mental set shifting

In a similar vein to belief updating, the right hemisphere has been shown to support mental set shifts. Cools et al. (2002) showed that the right vlPFC is active during the last trial before a hypothesis switch in the Wisconsin Card Sorting Test (WCST) and this activity is independent of the receipt of negative feedback (but *cf*. Konishi et al., 2002, who found left-lateralized activations for WCST set-shifting). Right hemisphere hypometabolism has also been linked to greater perseveration in the WCST (Lombardi et al., 1999; but *cf*. Stuss et al., 2000; Stuss and Alexander, 2007, who show perseveration in patient groups with both right and left hemisphere damage). Although there is mixed evidence, these results may suggest that the right hemisphere supports the shift from one hypothesis to another. If so, right hemisphere damage may cause beliefs to become fixed and immune to subsequent negative feedback.

Goel and Vartanian (2005) also highlight the role of the right vlPFC in set-shifting. They gave subjects problems that required the subjects to undergo lateral transformations—mental movements “from one state in a problem space to a horizontally displaced state rather than a more detailed version of the same state”—to correctly solve the problems (Goel and Vartanian, 2005, p. 1170). Based on the neuroimaging results and previous research, they concluded the right vlPFC initiates lateral transformations.

The importance of the right hemisphere in shifting from one mental set to another is also apparent in real-world tasks; Goel et al. (2013) asked patients with unilateral brain lesions to plan a trip to Italy and found that right hemisphere brain damaged patients made inferior plans compared with other patients and controls. Goel et al. (2013, p. 721) attribute the patients’ poor planning to substandard mental set shifting: “Damage to the right PFC system impairs the encoding and processing of more abstract and vague representations that facilitate lateral transformations. This results in prematurely locking onto precise concrete patterns, and quickly drawing conclusions, albeit substandard ones”.

### Monitoring and inhibition

Outside the domains of reasoning and hypothesis making, the right hemisphere has been shown to play a general role in monitoring and inhibiting thoughts and behavior. Chatham et al. (2012) and Stuss and Alexander (2007) review evidence that suggests that the right hemisphere, and specifically the right vlPFC, plays a role in context monitoring. Aron et al. (2014) propose that the right vlPFC serves as a brake to inhibit task-irrelevant behavior. Together, these general findings suggest that the right hemisphere monitors the environment for inconsistencies or goal-irrelevant stimuli and inhibits behaviors or thoughts that interfere with the goal at hand.

## Divergent hemispheric tendencies in reasoning

Based on the evidence presented above, we propose that the left and right hemispheres play different, yet complementary, roles in inferential reasoning. Evidence suggests that the left hemisphere tends to create inferences and explanations to resolve uncertainty. As Gazzaniga suggested over two decades ago, the left hemisphere is an interpreter (Gazzaniga, 1989). Its propensity to explain resembles abductive inference, or inference to the best explanation (Coltheart et al., 2010); its inferences do not necessarily have to be correct, or even plausible in some cases, as long as they bridge gaps in information and create a cohesive story. When free from the reign of an evaluative right hemisphere, the left hemisphere’s inferences may become excessive or inappropriate, and may fail to account for all contradictory evidence. However, this is not to say that the left hemisphere always makes poor explanations. Indeed, it seems as if the quality of the left hemisphere’s inferences is commensurate to the quality of the evidence it reasons with; most of the time, the left hemisphere’s inferences are sound and effectively reduce the uncertainty of the environment.

Unlike the left hemisphere, the right hemisphere, in our view, places a premium on the truth. It is sensitive to conflicts between hypotheses and real-world knowledge. In line with its roles in belief updating and set-shifting, we propose that the right hemisphere (1) monitors the plausibility of hypotheses and; (2) jettisons explanations that are inconsistent with reality or new evidence. Thus, the right hemisphere may prompt the revision of inappropriate hypotheses and initiate the search for new ones. In the realm of hypothesis making, if the left hemisphere is considered an interpreter, the right hemisphere may be considered a realist. While the left hemisphere strives to reduce uncertainty, the right hemisphere strives to reduce inconsistencies between hypotheses and reality.

Our conceptualizations of the left hemisphere as an interpreter and the right hemisphere as a realist are akin to Ramachandran’s distinction between left and right hemispheric tendencies (Ramachandran, 1996). According to Ramachandran, “The left hemisphere’s job is to create a model and maintain it at all costs. The right hemisphere’s strategy, on the other hand, is fundamentally different. I like to call it the ‘anomaly detector’, for when the anomalous information reaches a certain threshold, the right hemisphere decides that it is time to force the left hemisphere to revise the entire model and start from scratch” (pp. 351–352). These divergent tendencies to maintain vs. overhaul existing models are similar to Piaget’s concepts of assimilation (the propensity to integrate information into existing models) and accommodation (the propensity to modify existing models to better reflect reality) (Piaget, 1976). Although Piaget did not make any predictions concerning laterality, the evidence reviewed thus far suggests that the left hemisphere gravitates toward assimilation whereas the right hemisphere gravitates toward accommodation. Once the left hemisphere adopts a model that minimizes uncertainty and maximizes explanatory power, it incorporates new evidence into the model and may rationalize, ignore, or deny any contradictory evidence (Ramachandran, 1996). This “band-aid approach” creates a patchwork of explanations and rationalizations, but effectively reduces uncertainty by doing so. Unlike the left hemisphere, the neural processes clustered in the right hemisphere are sensitive to contradictions between beliefs and evidence. When contradictory evidence arises, the right hemisphere may prompt the reworking of the model to satisfy the new evidence or may suppress attempts at explanation altogether—in its view, it is better to be uncertain than wrong.

A study by Deglin and Kinsbourne (1996) offers a striking example of the hemispheres’ divergent reasoning strategies. In their experiment, patients undergoing electroconvulsive therapy (ECT) were shown two premises with familiar or unfamiliar content and were asked if a given conclusion supported or contradicted the premises. For example, patients were given the premises “Every state has a flag. Zambia is a state” and were asked “Does Zambia have a flag, or not?” Each patient completed the task on three separate occasions: once after ECT was used to suppress activity in the left hemisphere, once after ECT was used to suppress activity in the right hemisphere, and once before receiving ECT (which served as a control condition). Incredibly, even though the *same* syllogisms were presented to the *same* patients, the patients’ responses differed wildly depending on which hemisphere was suppressed. When patients’ right hemispheres were suppressed (and their left hemispheres were spared), their responses were consistent with the logic of the syllogism—even when the logic conflicted the patients’ beliefs and when the material was unfamiliar—and the patients responded faster and with more confidence, as compared to the control condition. In contrast, when the left hemisphere was suppressed (and the right hemisphere was spared) the patients tended to give responses that were consistent with real-world prior knowledge. Furthermore, if the premises contradicted real-world knowledge or were unfamiliar, the patients questioned the premises, answered with uncertainty, or refused to answer at all. For example, when presented with the Zambia syllogism, the *same subject* responded, “Each state has a flag, Zambia has also” under right hemisphere suppression but “Who knows it, this Zambia, how can I know whether it has a flag or not?” under left hemisphere suppression. This research supports our proposal of different hemispheric tendencies in reasoning: the left hemisphere readily makes inferences, regardless of content or conflicting evidence, and the right hemisphere ensures that conclusions are consistent with reality.

### Congruence with other hemispheric lateralization theories

Our framework is compatible with broader, more general theories of hemispheric lateralization. Bowden et al. (2005) propose that the hemispheres differ in the granularity of their computations and these differences produce distinct cognitive strategies and capabilities. Specifically, they suggest that computations in the right hemisphere are more coarsely tuned than those of the left hemisphere, an idea supported by the finding that the right hemisphere is more interconnected—on both a cellular and systems level—than the left hemisphere (Bowden et al., 2005). The relative coarse coding of the right hemisphere and relative fine coding of the left hemisphere would make the hemispheres predisposed to global and local processing, respectively. In the realm of inferential reasoning, the coarse, global processing of the right hemisphere may facilitate the detection of discrepancies between an explanation and its global, real-world context. Conversely, the finer coding of the left hemisphere may emphasize local cohesion at the expense of global consistency.

Braun’s “psychic tonus” model of hemispheric specialization is also consistent with our claim that the left hemisphere tends to create inferences while the right hemisphere tends to monitor, evaluate, and revise them (Braun, 2007). According to his model, the left hemisphere generally activates mentation and behavior while the right hemisphere inhibits them. More colloquially, the left hemisphere is predisposed to “do something” and the right hemisphere is inclined to “freeze and recoup” (Braun, 2007, p. 418). The propensity of the left hemisphere to act and create is consistent with our view that it plays a prominent role in making inferences and bridging gaps in information. Likewise, the right hemisphere’s tendency to inhibit behavior is in line with our proposal that it plays a role in monitoring and inhibiting inappropriate hypotheses.

In his review, Braun also suggests that the hemispheres act in opposition to each other: the suppression of cognitive modules in one hemisphere activates their counterparts in the other hemisphere and vice versa. Craig (2005) echoes the idea of hemispheric opposition in his review of emotional asymmetry and suggests that the balance between opposing hemispheric systems facilitates homeostasis and health. It is possible that hemispheric opposition also plays a role in inferential reasoning; the two hemispheric reasoning systems may compete during inference making and may be preferentially recruited in accordance with situational demands. A dual reasoning system with opponent interactions could promote balanced and flexible inference making. Moreover, disruption in the balance between the two component systems may explain aberrant reasoning in brain-damaged and delusional patients. In the case of right hemisphere brain damage, an overactive left-lateralized reasoning system may lead to excessive inference making. Conversely, a right-lateralized reasoning system resulting from left hemisphere brain damage may lead to insufficient inference making, as patient studies suggest.

In his theory of hemispheric lateralization, Corballis (1989) proposes that the left hemisphere is uniquely generative; that is, it combines visual, lexical, or semantic elements to create “novel assemblages” (p. 499). Corballis argues that the left hemisphere’s capacity for language and visual image production is rooted in its ability to create novel combinations. In the realm of reasoning, the generativity of the left hemisphere could facilitate, or perhaps even underlie, inference making, since both processes involve making new associations to create a cohesive whole. Unlike the left hemisphere, Corballis suggests that the right hemisphere uses an analog code of representation; that is, its neural representations reflect reality and leave less room for interpretation and manipulation. Again, the right hemisphere’s tendency to represent items veridically is consistent with our view that the right hemisphere values truth and consistency during reasoning.

Finally, in their review of the functional anatomy of inferential reasoning, Barbey and Patterson (2011) present evidence that the vlPFC supports explanation generation, the dorsolateral PFC supports explanation evaluation, and the anterior PFC supports the integration of inferences. Although they did not emphasize hemispheric specialization in the review, their meta-analysis reveals a distinct hemispheric lateralization, such that the left hemisphere supports hypothesis formation and integration and the right hemisphere supports hypothesis evaluation. These findings are consistent with our view that the left hemisphere plays a prominent role in forming inferences and the right hemisphere plays a prominent role in monitoring the validity of inferences.

## Problems and challenges

This review focused on higher-order inferential reasoning and surveyed tasks involving reasoning problems that were complicated and semantic in nature. In other domains of causal inference making, such as comprehension or perception, the left hemisphere vs. right hemisphere distinction we present here may not hold. For example, although the left hemisphere is generally superior at making judgments about causal structure, the right hemisphere has been shown to skillfully make perceptual (Corballis, 2003; Miller and Valsangkar-Smyth, 2005; Roser et al., 2005) and possibly social (Wende et al., 2013) causal judgments and inferences. The right hemisphere may also play a prominent role in processes related to inference making, such as solving problems with insight (Bowden and Jung-Beeman, 2003; Kounios and Beeman, 2014) or comprehending natural language (Jung-Beeman, 2005). However, it is possible that additional constraints on each hemisphere, for which we currently have only oblique evidence, might resolve some of these ambiguities. For instance, the right hemisphere might value parsimony to a greater degree than the left, and may therefore be more likely to arrive at the correct solution in problems requiring insight (or rather, less likely to arrive at convoluted, incorrect solutions).

According to our claim that the right hemisphere monitors the validity of inferences, a damaged or disconnected right hemisphere should lead to implausible or excessive inference making and possibly even delusions. However, split-brain patients and some patients with unilateral right hemisphere brain damage fail to develop delusional disorders (Gazzaniga, 2000; Coltheart, 2010). Coltheart’s two factor theory of delusions may offer an explanation as to why split-brain patients and patients with right hemisphere brain damage do not succumb to disorders in reasoning. As we stated earlier, Coltheart proposes that delusions require: (1) an impairment that elicits some sort of explanation and; (2) a dysfunctional belief evaluation system that fails to reject implausible explanations for the initial impairment (McKay et al., 2007; Coltheart, 2010; Coltheart et al., 2010, 2011). We suggest that split-brain patients and unilateral right brain-damaged patients may meet the second requirement but not the first. That is, these patients may have an impaired evaluation system but they fail to develop delusional beliefs because they lack a neurological impairment that warrants an explanation. Right hemisphere damage or isolation may therefore make a patient vulnerable to abnormal reasoning, but it does not necessarily precipitate delusional ideation; in other words, left hemisphere over-activity or right hemisphere under-activity is necessary, but not sufficient, for delusions to form. It is also likely that—although the right hemisphere plays a leading role in hypothesis evaluation—the left hemisphere retains some evaluative capabilities, which may prevent delusional beliefs from forming.

Although several lines of evidence support our framework, some do not. Under our framework, the receipt of inconsistent evidence should preferentially activate regions in the right hemisphere, but Fugelsang and Dunbar (2005) found left-lateralized activations in subjects who were presented with evidence that conflicted a theory. Since this study utilized a block design, however, it is possible that it failed to capture the true neural activity associated with the presentation of contradictory evidence; the left lateralized activations may instead reflect inferential reasoning processes other than hypothesis evaluation and belief updating. A study by Vartanian and Goel (2005) also contradicts our predictions. They propose that the right vlPFC supports unconstrained hypothesis generation, since they found that activity in the right vlPFC increases as the constraints of an anagram-solving task decrease. An alternative explanation for their results could be that the right vlPFC plays a role in monitoring the problem solving process or inhibiting unfruitful lines of reasoning, both of which would fit within our framework.

The strongest evidence against our proposal stems from two transcranial magnetic stimulation (TMS) studies conducted by Tsujii et al. (2010, 2011). In both studies, TMS was applied to the left and right inferior frontal cortices of subjects prior to a deduction task that involved belief-logic conflicts. Since we postulate that the right hemisphere favors truth and real-world knowledge over logic, we would expect inhibition of the left vlPFC, but not the right vlPFC, to cause an over-reliance on prior beliefs. Contrary to our predictions, they found that right vlPFC disruption increased the influence of prior beliefs on logic (thus enhancing the belief-bias effect) and left vlPFC disruption eliminated the belief-bias effect. It is possible that the right hemisphere’s role in reasoning is not only to monitor, revise, and reject improbable hypotheses, as we have proposed, but also to inhibit information that is irrelevant to reasoning. In this case, disruption of an inhibitory right vlPFC would allow conflicting prior beliefs to bias reasoning, thus enhancing the belief-bias effect as Tsujii et al. (2010, 2011) found. Alternatively, it is possible that the targeted brain regions were not modulated as intended, even though the stimulation parameters used in the studies have been shown to reduce cortical excitability in motor cortex following stimulation (Robertson et al., 2003). In any case, additional studies using complementary neuromodulation techniques are needed to corroborate and expand on these findings.

Finally, there are general caveats to interpreting evidence from various lines of research. For instance, lesion and psychiatric data come from studies of the abnormal brain, which both adds additional noise (no two lesions are identical) and gives room for a host of compensatory mechanisms to influence behavior. Conversely, neuroimaging fails to ascribe causality to brain activity in any particular region—that is, given that both hemispheres presumably process incoming events, higher activity in one hemisphere than the other may simply reflect additional *effort* (i.e., less efficient or effective processing) on the part of the more active hemisphere, rather than its necessity for performance. Carefully designed studies (that correlate activity with accuracy, for instance) can help allay these concerns. Neuroimaging studies may especially fail to shed light on hemispheric lateralization: contrasts designed to find the neural correlates of one process may be contaminated by the neural fingerprints of another, which may in turn make a truly lateralized process appear bilateral. We also necessarily make the neuroimager’s fallacy (Poldrack et al., 2008) in almost every case, as the vast majority of studies—even those that report lateralization results—only report clusters of activation, not direct contrasts across hemispheres.

## Discussion

In this review, we present converging lines of evidence from research on split-brain, delusional, brain-damaged, and healthy subjects that suggest the left hemisphere creates hypotheses and the right hemisphere evaluates these hypotheses and rejects them if they conflict with reality. Similarly, it appears the left hemisphere is driven toward reducing uncertainty, and does so by creating inferences to bridge information gaps, and the right hemisphere is driven toward reducing conflict, and does so by revising and rejecting explanations that are inconsistent with reality. In a healthy human brain, these divergent hemispheric tendencies complement each other and create a balanced and flexible reasoning system. Working in unison, the left and right hemispheres can create inferences that have explanatory power and both internal and external consistency.

### Functional localization considerations

So far we have only considered the lateralization of hypothesis formation and evaluation. Although the specific functional localization of these processes is outside the scope of this paper, we would like to briefly comment on potential sites of hypothesis making in the brain. Several studies indicate that the right lateral prefrontal cortex, and more specifically the right vlPFC, is recruited during hypothesis evaluation. Activity in the right vlPFC is associated with effortful inhibition (Aron et al., 2014), detecting and resolving conflicts between beliefs and logic (Goel et al., 2000; Goel and Dolan, 2003; Goel, 2007; Menenti et al., 2009; Stollstorff et al., 2012), and rejecting inappropriate hypotheses in accordance with new evidence (Cools et al., 2002). Conversely, the left vlPFC and left dorsomedial PFC have been linked to hypothesis formation. These areas are consistently activated during deduction (Goel et al., 2000; Friese et al., 2008; Rodriguez-Moreno and Hirsch, 2009; Monti and Osherson, 2012) and have been shown to be active during reasoning about autobiographical (D’Argembeau et al., 2013) or social (Barbey and Patterson, 2011) events.

## Conclusion

We would like to emphasize that we are proposing the hemispheres have different reasoning *propensities*. We do not claim these different reasoning styles generalize to all individuals and all reasoning problems, nor do we claim that the processes we discuss in this review are completely lateralized to one hemisphere or the other. The right hemisphere may make inferences to some extent (especially in certain cognitive domains) and the left hemisphere may possess some evaluative capabilities. Indeed, the facts that split-brain patients do not develop bizarre, pathological beliefs and delusional patients occasionally experience intermittent symptoms (Coltheart, 2010) suggest that hypothesis formation and evaluation are not totally lateralized in the brain. However, a large body of evidence suggests that the natural reasoning tendencies and strategies of the hemispheres do differ. Cognitive processes in the left hemisphere *tend* to make inferences while those in the right hemisphere *tend* to detect and correct inconsistencies. These findings are consistent and robust among patient studies and, to some extent, among neuroimaging studies using healthy participants.

Our framework offers several testable predictions. First, neuroimaging studies using tasks that isolate hypothesis formation or hypothesis evaluation should find increased brain activity in the left and right hemispheres, respectively. However, it may prove difficult to isolate the brain processes underlying only one subcomponent of inferential reasoning since healthy individuals naturally form, monitor, and evaluate inferences concurrently. This may obfuscate attempts to identify the neural basis of inferential reasoning processes and may make lateralized processes appear bilateral. Neuromodulation with TMS or transcranial direct current stimulation (tDCS) may provide a promising alternative. By testing subjects’ reasoning abilities and shortcomings under unilateral hemispheric suppression or stimulation, it may be possible to determine the unique roles each hemisphere plays in inferential reasoning. We predict subjects under right hemisphere suppression (and perhaps left hemisphere stimulation) will retain the ability to make inferences but may be prone to accept implausible explanations. Conversely, we predict subjects under left hemisphere suppression (and perhaps right hemisphere stimulation) will retain the ability to detect conflicts or inconsistencies in reasoning, but may be impaired in making inferences and creating explanations. We predict similar reasoning biases to be present in other cases of hemispheric asymmetry in the frontal lobes (such as functional asymmetries arising from neurological conditions, pharmaceutical manipulations, or simply normal brain development and aging). However, we expect that these biases will be weaker and less consistent than those observed in patients with brain damage or participants undergoing neuromodulation since changes in hemispheric dominance may be subtle and accompanied by a host of other neural changes and compensatory mechanisms.

Lateralized processing in the brain is both prevalent and beneficial: it maximizes the use of cortical space by reducing redundancy (Corballis, 1989; Gazzaniga, 2000; Vallortigara, 2006; Hopkins and Cantalupo, 2008), enhances brain efficiency by supporting dual processing (Rogers, 2000; Rogers et al., 2004; Vallortigara, 2006), reduces interhemispheric conflict by allowing the hemispheres to operate in separate problem spaces (Corballis, 2003; Vallortigara, 2006), and increases processing speed (Ringo et al., 1994). Hemispheric lateralization of inferential reasoning may be especially beneficial. Dual reasoning strategies—one driven to reduce uncertainty and the other driven to resolve inconsistency—create a flexible, efficient, and balanced reasoning system. Cognitive modules in the left hemisphere, with their propensities to create explanations, bridge gaps, and infer causation, may be preferentially recruited in situations that require creativity and liberal inference making. Conversely, cognitive modules in the right hemisphere, with their tendencies to detect conflict, monitor explanations in a global context, and inhibit inappropriate inferences, may play a greater role in situations that necessitate caution and conservative reasoning. Simply biasing these reasoning systems in accordance with situational demands ensures reasoning is adaptive, sensible, and efficient. In a healthy brain, the different inferential capabilities of the hemispheres enhance reasoning by maximizing both explanatory power and plausibility.

## Conflict of interest statement

The authors declare that the research was conducted in the absence of any commercial or financial relationships that could be construed as a potential conflict of interest.
